# Cerebral Vasospasm in Patients over 80 Years Treated by Coil Embolization for Ruptured Cerebral Aneurysms

**DOI:** 10.1155/2014/253867

**Published:** 2014-03-24

**Authors:** Tomohito Hishikawa, Yuji Takasugi, Tomohisa Shimizu, Jun Haruma, Masafumi Hiramatsu, Koji Tokunaga, Kenji Sugiu, Isao Date

**Affiliations:** Department of Neurological Surgery, Okayama University Graduate School of Medicine, Dentistry and Pharmaceutical Sciences, 2-5-1 Shikata-cho, Kita-ku, Okayama City, Okayama 700-8558, Japan

## Abstract

*Object*. The effect on clinical outcomes of symptomatic vasospasm after aneurysmal subarachnoid hemorrhage (SAH) in patients over 80 years who underwent coil embolization was evaluated. *Methods*. Forty-four cases were reviewed and divided into two groups according to patient age: Group A, 79 years or younger, and Group B, 80 or older. Patient characteristics, prevalence of symptomatic vasospasm, modified Rankin Scale (mRS) scores at discharge and frequency of symptomatic vasospasm in patients with mRS scores of 3–6 were analyzed. *Results*. Thirty-two (73%) of the 44 cases were categorized as Group A and 12 (27%) as Group B. Group B had a significantly higher prevalence of symptomatic vasospasm compared to Group A (*P* = 0.0040). mRS scores at discharge were significantly higher in Group B than in Group A (*P* = 0.0494). Among cases with mRS scores of 3–6, there was a significantly higher frequency of symptomatic vasospasm in Group B than in Group A (*P* = 0.0223). *Conclusions*. In our cohort of aneurysmal SAH patients treated by coil embolization, patients over 80 years of age were more likely to suffer symptomatic vasospasm, which significantly correlated with worse clinical outcomes, than those 79 years and under.

## 1. Introduction

The numbers of patients over 80 years of age with subarachnoid hemorrhage (SAH) due to rupture of cerebral aneurysms are increasing. According to the Japanese Stroke Data Bank, a database on acute stroke patients with the aim of furthering the standardization of stroke management in Japan, about 10% of SAH patients in 2009 were over 80 years of age. The International Subarachnoid Aneurysm Trial (ISAT) revealed the superiority of coiling to clipping for ruptured cerebral aneurysms in terms of clinical outcomes [1]. Coil embolization is also less invasive, and its advantages for elderly patients have already been reported [2–5]. Together, these findings are increasing the popularity of coil embolization and also the likelihood that a patient treated for ruptured cerebral aneurysm will undergo coil embolization.

Cerebral vasospasm is the most common cause of focal ischemia after SAH and has been reported to be one of the important causes of death and disability among SAH patients [6]. A few papers have investigated the effect of age on the occurrence of cerebral vasospasm in surgically treated cases [7, 8]. In the present study, we evaluated the effect of vasospasm on outcomes in patients who had undergone coil embolization for ruptured cerebral aneurysm at our institute, comparing patients over 80 years of age with those 79 years and under.

## 2. Patients and Methods

### 2.1. Patients

We reviewed the clinical information of all patients admitted to Okayama University Hospital with acute aneurysmal SAH from January 2003 to May 2012. We included 88 patients who met the following inclusion criteria: (1) SAH as demonstrated on computed tomography (CT); (2) evidence for ruptured intracranial aneurysm as demonstrated by cerebral angiography or 3-dimensional CT angiography; and (3) aneurysm treated with surgical clipping or endovascular coiling. Decisions regarding aneurysm treatment modality were made on the basis of factors including aneurysm location, size and shape, patient age, neurological grading (Hunt and Kosnik grade) [9], and Fisher group [10]. Finally, the medical records of 44 aneurysmal SAH patients treated by coil embolization were evaluated retrospectively. Two patients experienced two episodes of SAH during this period. Only the first SAH of these two patients was included and a total of 44 aneurysmal SAH cases were analyzed in this study. These cases were divided into two groups, according to patient age: Group A, 79 years or younger, and Group B, 80 years or older.

### 2.2. Endovascular Procedures

All endovascular procedures but one were performed under general anesthesia. The simple technique was used in 23 cases (52%); the other 21 (48%) were treated using adjunctive techniques as follows: balloon remodeling technique (BRT): 15 cases (34%), double catheter technique (DCT): 3 cases (7%), BRT combined with DCT: 3 cases (7%). Two senior neurosurgeons (K. Sugiu and K. Tokunaga) performed all endovascular procedures.

### 2.3. Management of Cerebral Vasospasm

All cases were maintained in a normotensive and normovolemic state and treated with thromboxane A_2_ synthesis inhibitor and fasdil chloride intravenously starting immediately after coil embolization. After the diagnosis of symptomatic vasospasm had been reached, mild hypertensive hypervolemic therapy was initiated. Lumbar drainage was indicated for the patients assigned to Fisher group 3 and inserted in 36 cases (82%) to remove subarachnoid clots. Symptomatic vasospasm was defined, according to Shirao et al. [11], as (1) the presence of neurological worsening including focal deficit, decline in level of consciousness, and motor paresis; (2) no other identifiable cause of neurological worsening; (3) confirmation of vasospasm by medical examinations including evidence of vasospasm by radiographic assessment. In our institute, symptomatic vasospasm was confirmed by angiography and subsequently treated with endovascular intra-arterial fasdil chloride administration and/or angioplasty. To identify any instances of cerebral infarction, patients with symptomatic vasospasm were repeatedly checked by CT scanning. There were no differences of the treatment for vasospasm between the groups.

### 2.4. Neurological Evaluation

A neurosurgeon in our institute assessed the neurological status of all patients using the modified Rankin Scale (mRS) [12] at the time of hospital discharge.

### 2.5. Statistical Analysis

Quantitative variables are presented as percentages or as medians and interquartile ranges (IQRs). Statistical analysis was performed using Fisher's exact probability test, the chi-square test, and the Mann-Whitney *U* test, as appropriate. All statistical analyses were performed using StatView (SAS Institute, Cary, NC, USA). Differences were considered to be significant when *P* values were less than 0.05.

## 3. Results

Thirty-two (73%) of the 44 cases were categorized as Group A and 12 (27%) as Group B.  Table 1 shows the patient characteristics of the cases in this study. There was no statistically significant difference related to patient sex or treatment day. Group B had a tendency toward more anterior circulation aneurysms compared to Group A, but this was not significant (*P* = 0.0679). Fisher groups and Hunt and Kosnik grades were evenly distributed in Groups A and B.

### 3.1. Prevalence of Symptomatic Vasospasm and Cerebral Infarction

The frequency of insertion of lumbar drainage was significantly higher in Group A than in Group B (*P* = 0.0249). Four of the 32 cases (13%) in Group A and 7 of the 12 cases (58%) in Group B exhibited symptomatic vasospasm. The difference in the prevalence of symptomatic vasospasm between Groups A and B was statistically significant (*P* = 0.0040). Group B had a significantly higher prevalence of cerebral infarction due to vasospasm compared to Group A (33% versus 3%, *P* = 0.0153).

### 3.2. Outcomes at Discharge

mRS scores at discharge were significantly higher in Group B than in Group A (median 4 [IQR 3.75–4.25] versus 3 [IQR 1.75–4], *P* = 0.0494, Table 1). There was no significant difference between Groups A and B in length of hospital stay (Table 1).

### 3.3. Effect of Symptomatic Vasospasm on Outcomes

Table 2 shows the prevalence of symptomatic vasospasm in patients with mRS scores of 3–6 at discharge in Groups A and B. Four of 23 cases (17%) with mRS scores of 3–6 in Group A and 7 of 12 cases (58%) with mRS scores of 3–6 in Group B exhibited symptomatic vasospasm. There was a significant difference between Groups A and B in the frequency of symptomatic vasospasm in cases with mRS scores of 3–6 (*P* = 0.0223).

## 4. Discussion

### 4.1. Aging and SAH

As Japan has the world's highest life expectancy, aging in stroke patients has become an important social issue from the perspective of health care and medical economy in Japan. The incidence of SAH increases with age, and this tendency is of peculiar note given the recent aging of the population. Inagawa [13] reported that the percentage of very elderly SAH patients over 80 years in Izumo city, Japan, increased from 5% in 1980–1989 to 18% in 1990–1998. It has also been reported that the size of ruptured aneurysms tends to increase with age [14]. Considering the larger size of aneurysms and the likely presence of comorbid disease in patients over 80 years, determining optimal management plans for these patients is difficult, and the prognosis of elderly SAH patients has been reported to be poor [8]. Recently, coil embolization is becoming a more common treatment for aneurysmal SAH patients, especially elderly patients, as this treatment has been proven to contribute to better clinical outcomes in comparison with clipping in a large randomized clinical trial [1]. It would be helpful to elucidate the outcomes of aneurysmal SAH patients over 80 years of age who have been treated by coil embolization, but few studies have analyzed outcomes in this population exclusively. This is the first report examining the effect of vasospasm on clinical outcomes in patients over 80 years of age treated by coil embolization.

### 4.2. Significance of Vasospasm in Elderly SAH Patients

This study revealed that clinical outcomes at discharge were significantly worse in patients over 80 years than in patients 79 years and under. The overall prevalence of symptomatic vasospasm was significantly higher in patients over 80 years than in patients 79 years and under (58% versus 13%). In addition, the frequency of symptomatic vasospasm in patients with poor outcome (mRS scores 3–6) was significantly higher in patients over 80 years than in patients 79 years and under (58% versus 17%).

Several mechanisms could be responsible for the higher prevalence of symptomatic vasospasm among elderly patients. First, aging itself could be related to the higher prevalence of symptomatic vasospasm in elderly patients. Inagawa [15] demonstrated that angiographical vasospasm in major cerebral vessels tended to be lower in the elderly because of good clearance of the accumulated blood clots with cerebrospinal fluid or atherosclerotic changes of arteries. Other papers have reported that elderly patients developed slightly but significantly fewer angiographical vasospasms than younger patients did, although they tolerated ischemia less well and were prone to develop infarction [7, 8]. In fact, our data reveal a significantly higher prevalence of cerebral infarction in patients over 80 years of age than in patients of 79 years and under. Together with the data from previous reports [7, 8, 15], our data indicates that there is a discrepancy between angiographical vasospasm and cerebral ischemia in elderly patients; our data also raise the possibility that the discrepancy could be attributable to the vulnerability of the aged brain. This vulnerability of the aged brain to vasospasm is probably explained by the reduction of cerebral blood flow or the impairment of vascular reserve in elderly SAH patients [16, 17]. Especially in patients over 80 years of age, aging has a profound effect on symptomatic vasospasm and subsequent cerebral infarction. Second, the effect of lumbar drainage on vasospasm should be considered. Klimo Jr. et al. [18] reported that shunting of cerebrospinal fluid through a lumbar drainage after SAH markedly reduced the risk of vasospasm and improved outcomes. The lower frequency of insertion of lumbar drainage in patients over 80 years in this investigation might be related to the higher prevalence of symptomatic vasospasm. It is generally supposed to be more difficult to insert lumbar drainage in patients over 80 years of age in the clinical setting because they sometimes have significant lumbar spine degeneration.

The overall correlation of poor outcome with advancing age has been partially explained by some factors, such as mRS scores at onset [19], brain atrophy [20], poor neurological grade at admission [21], increased amount of blood on CT, and preexisting medical diseases [8]. The greater incidence of poor outcome in elderly patients is multifactorial, and symptomatic vasospasm is probably just one of the factors involved in poor outcome. Of the various factors that can be involved, vasospasm is significant as the only pathophysiology that can be overcome through successful perioperative management. Cerebral vasospasm even in SAH patients over 80 years of age should be treated more aggressively and earlier to improve clinical outcomes.

### 4.3. Study Limitations

There are certain limitations to this study that should be noted. First, this study had a retrospective nature and a limited sample size. A larger population of SAH patients is needed to build the accurate evidence on the effect on clinical outcomes of symptomatic vasospasm in patients over 80 years who underwent coil embolization. Second, the outcomes of patients over 80 years of age in our investigation were worse than those in some previous reports [22, 23]. This is probably because the length of hospital stay was relatively short (median 32.5 days) and outcomes were therefore evaluated relatively early. A longer follow-up in a future study is warranted because some patients could improve their mRS scores with the aid of rehabilitation. Third, the patients in this study were not randomized; rather, there was a selection bias involving in determining treatment modality. Patients over 80 years of age tended to have more anterior circulation aneurysms compared to patients 79 years and under in this investigation. Yet there is no definitive evidence of a significant relationship between aneurysm location and symptomatic vasospasm [24, 25]. The difference between the groups in aneurysm location probably had little influence on the occurrence of symptomatic vasospasm.

## 5. Conclusions

In our patient cohort, aneurysmal SAH patients treated by coil embolization who were over 80 years of age were more likely to suffer symptomatic vasospasm compared to those of 79 years and under. In patients over 80 years, symptomatic vasospasm was significantly correlated with worse clinical outcomes. The development of an improved treatment for vasospasm could help improve clinical outcomes in SAH patients over 80 years of age.

## Figures and Tables

**Table 1:** Patient characteristics.

	No. of cases (%)		*P* value
	Group A (32 cases)	Group B (12 cases)	
Age, yr (median, IQR)	64 (58.75–69.75)	83 (82–84.25)	
Sex (male : female)	12 : 20	6 : 6	0.4526
Treatment day (median, IQR)	0 (0-1)	0 (0-1)	0.9877
Location of aneurysms (anterior circulation)	19 (59)	11 (92)	0.0679
Fisher groups			0.1158
2	2 (6)	3 (25)	
3	30 (94)	9 (75)	
Hunt and Kosnik grades			0.4752
I	3 (9)	3 (25)	
II	8 (25)	3 (25)	
III	11 (35)	2 (17)	
IV	10 (31)	4 (33)	
Insertion of lumbar drainage	29 (91)	7 (58)	0.0249
Symptomatic vasospasm	4 (13)	7 (58)	0.0040
Cerebral infarction	1 (3)	4 (33)	0.0153
Length of stay, days (median, IQR)	30 (17–53)	32.5 (14.75–50.25)	0.7515
mRS score at discharge			0.0494
0	2 (6)	0 (0)	
1	6 (19)	0 (0)	
2	1 (3)	0 (0)	
3	9 (28)	3 (25)	
4	9 (28)	6 (50)	
5	5 (16)	3 (25)	
6	0 (0)	0 (0)	

Age, treatment day, and length of stay values represent medians (interquartile range); other values represent raw numbers with percentages in parentheses.

mRS indicates modified Rankin Scale.

**Table 2:** Prevalence of symptomatic vasospasm in patients with mRS score of 3–6 at discharge.

	Group A (23 cases)	Group B (12 cases)	*P* value
Symptomatic vasospasm			
Yes	4 (17)	7 (58)	0.0223
No	19 (83)	5 (42)	

Values represent raw numbers with percentages in parentheses.
